# Effects of Attrition Shoes on Balance Control Ability and Postural Stability Following a Single-Leg Drop Jump Landing

**DOI:** 10.3390/healthcare11081127

**Published:** 2023-04-14

**Authors:** Shane-Fei Chen, Yan Wang, Yinghu Peng, Ming Zhang

**Affiliations:** 1 Department of Biomedical Engineering, Faculty of Engineering, The Hong Kong Polytechnic University, Hong Kong 999077, China; 2 Hong Kong Polytechnic University Shenzhen Research Institute, Shenzhen 518057, China; 3 Research Institute for Sports Science and Technology, The Hong Kong Polytechnic University, Hong Kong 999077, China

**Keywords:** worn shoes, time to stabilization, single-leg drop jump, dynamic balance, fall injury

## Abstract

The purpose of the study is to determine the influence of lateral-heel-worn shoes (LHWS) on balance control ability through the single-leg drop jump test. The results could be beneficial by preventing lower limb injuries. Eighteen healthy participants performed the single-leg drop jump test. Times to stabilization for ground reaction forces (TTSG) in the anterior/posterior, medial/lateral, and vertical directions were calculated to quantify dynamic balance control ability. Outcome variables of the center of pressure (COP) were used to examine the main effect of LHWS during the static phase. The postural control ability was assessed through time to stabilization for the center of mass (TTSC) in the three directions. TTSG and TTSC for the LHWS group were found to be longer than those for the new shoes (NS) group in the M/L direction (*p* < 0.05). An increase in the TTS revealed an increased risk of falls during physical activities. However, no significant effects for both TTSG and TTSC were found in the other two directions between LHWS and NS groups. A static phase was cropped using TTSG for each trial, which indicated a phase after participants obtained balance. Outcome measures derived from COP showed no significant effects in the static phase. In conclusion, LHWS weakened balance control ability and postural stability in the M/L direction when compared to the NS group. During the static phase, no significant differences were found between the LHWS group and the NS group in balance control ability and postural stability. Consequently, lateral-worn shoes might increase the risk of fall injuries. The results could serve as an evaluation of shoe degradation for individuals with the aim of avoiding the risk of falls.

## 1. Introduction

Footwear plays an important role in balance control ability, which is essential in the prevention of injuries in a weight-bearing condition [1]. Individuals’ footwear might wear after a long time of wearing. Inappropriate footwear was indicated to be associated with bone fractures [2]. Additionally, high correlations were investigated between the outsoles of footwear and balance [3]. Worn shoes may attenuate balance control ability, thus increasing the risk of falls [4] and ankle sprains during physical activities [5]. A previous study has indicated that the degradation of shoes is one of the significant risk factors for running-related injuries [6]. The influence of shoe degradation on biomechanics during running were examined [2]. As for the worn-shoe group, the stance time increased significantly and the ankle revealed a decrease in the maximum dorsiflexion, which indicated that runners might alter their running patterns to maintain constant external loads. Another study [7] used a 1 mm thick tapered wedge to simulate the wear of footwear. The results revealed consistent results that lateral heel wear led to a decrease in functional performance under the condition of single-leg weight bearing. Moreover, the greater size of the shoe-worn region was also indicated to increase slip occurrence during walking for healthy adults [8]. Worn shoes might alter the biomechanics of individuals during activities [9]. Understanding the biomechanical effects of worn shoes on balance control ability could enhance individuals’ attention to shoe attrition, which could reduce the injuries induced by worn shoes. 

Dynamic balance is defined as the ability to maintain or recover balance while performing movements [10]. One technique to assess dynamic stabilization is a jump landing task that has been proposed to have consistent sensitivity for instability [11]. The single-leg drop jump landing test has often been used to examine balance control ability [12], which is complex and sports-related [13]. The test assesses the balance control transition from a dynamic phase to a static state [14]. Participants need to jump from a platform and land on a force platform. As the participants are required to jump from a platform with a certain height, the GRF in the vertical and anteroposterior are large enough to differentiate different conditions [12]. Consequently, the tests could be regarded as an appropriate approach to detect impairments in dynamic balance [15]. Outcome measures from ground reaction forces (GRF) were used mostly to quantify the test performance [16]. Time to stabilization (TTS) was calculated to assess balance ability, which is the time that the GRF in a given direction stabilizes within the static baseline following a single-leg drop jump [17]. The dynamic stability was examined for healthy individuals with the calculation of TTS under different footwear conditions using this test in previous studies [18]. The sequential averaging approach is one of the most frequently used methods to calculate TTS, the value of which reflects balance control ability during the dynamic phase [19,20]. On the other hand, single-leg standing balance during the static phase is usually assessed using the center of pressure (COP) measured by force platforms, which is considered to be the gold standard measure [21]. The outcome measures derived from COP could discriminate static postural stability effectively [22]. However, these measures only provide the estimation of stability control without the consideration of postural balance [23].

Footwear could alter the posture of the feet and the ground contact and/or influence several sensory systems, which importantly contribute to postural control [24]. Sole Christopher Charles, et al. [25] revealed that skew footwear might impair postural stability. The adaptation of posture might cause a decrease in the ability to recover from perturbations [25]. The decrease in postural stability was indicated to increase the risk of falls, especially for older adults [26]. During a single-leg drop jump landing task, the jump could be considered a balance disturbance, and the recovery of balance stabilization is the postural control pursuit after the impact on the force platform [27]. This spatial stabilization is usually evaluated by outcome measures of the center of mass (COM) [28]. Since outcome measures from GRF and COP could not assess postural stability, outcome variables derived from COM were adopted to assess the postural responses to disturbance [29].

The degradation of shoes is inevitable for individuals in daily life. The degradation of footwear was also indicated to significantly contribute to injuries [30]. The differences in shoe support could be associated with balance [31]. With the wearing time of footwear increasing, the degeneration mostly appears at the lateral heel [32]. This worn pattern leads to lateromedial asymmetry in the heel, the effects of which on balance control stabilization have been seldom studied. Therefore, it is hypothesized that lateral-heel-worn shoes (LHWS) impair balance control ability and posture stability. The single-leg drop jump test was conducted to examine the influences. Outcome measures from GRF and COM were analyzed to reveal the influence. 

## 2. Methodology

### 2.1. Participants

A sample size estimation was performed with a significance level of 0.05 and a statistical power of 0.8 in Gpower (G*power 3.0.10, Universität Düsseldorf, Düsseldorf, Germany). The minimum sample size was calculated to be 15 participants with a medium effect size of 0.8. A convenience sample of 18 healthy participants (12 males, 6 females, age = 25.3 ± 5 years, weight = 69.5 ± 12.2 kg, and height = 173.6 ± 9.3 cm) was recruited from Hong Kong Polytechnic University. All participants were free of lower limb musculoskeletal injuries. In addition, abnormal foot morphology was also excluded from participant recruitment in this study. As individuals might have different degradation types of worn shoes due to their gait patterns, all the participants recruited in the current study were confirmed to have lateral-heel-worn patterns using their previous shoes. Informed written consent was obtained by all participants before the experiment. This study was approved by the Human Subjects Ethics Sub-Committee of Hong Kong Polytechnic University (number: HSEARS20150121003).

### 2.2. Footwear Conditions

**Figure 1:**
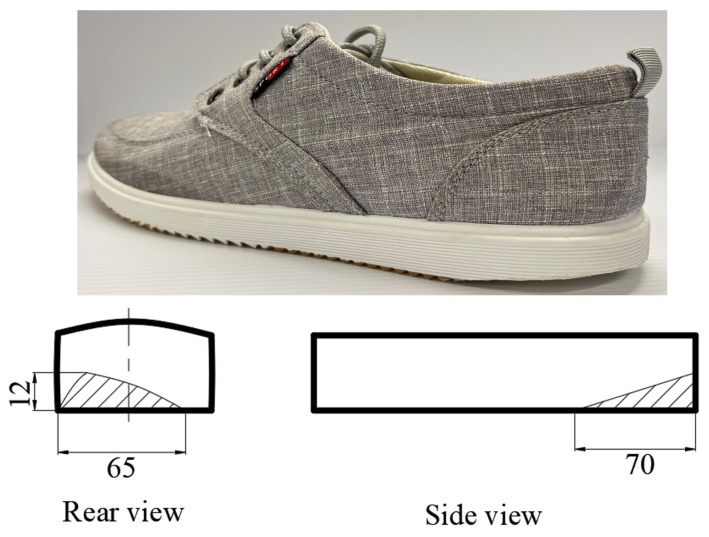
Schematic diagram of LHWS outsoles. LHWS: lateral-heel-worn shoes.

The current study adopted common canvas shoes to investigate the impact of worn shoes on balance ability. Participants wore laboratory-provided shoes with two conditions that fit their sizes to perform the experiments. These two conditions of the shoes were the same for all the participants. A schematic diagram of the worn shoe with lateral attrition heel (LHWS) is shown in Figure 1. As for the degradation severity of shoes, it might be a little different for individuals due to the walking pattern and wearing time. The severity of the worn shoes was defined by the worn length, worn width, and worn thickness, as was adopted by a previous study [33]. A previous study recruited seventy-six subjects to perform shoe abrasion tests with a 14-week training [32]. The results indicated that the maximum worn volume was almost 9 cm^3^ in the posterolateral heel and that the maximum progression angle was over 10 degrees. Therefore, the current study adopted the measurements of the worn parts to indicate the severity of the worn shoes. As can be seen in the schematic diagram of the worn shoes shown in Figure 1, the worn severity was quantified by the maximum length, which is 70 mm in the anterior–posterior (A/P) direction, the maximum width, which is 65 mm in the medial–lateral (M/L) direction, and the maximum height, which is 12 mm in the vertical direction. All participants performed the test in shoes with equal severity of wear to examine the immediate effects of LHWS. 

### 2.3. Single-Leg Drop Jump Landing Protocol

**Figure 2:**
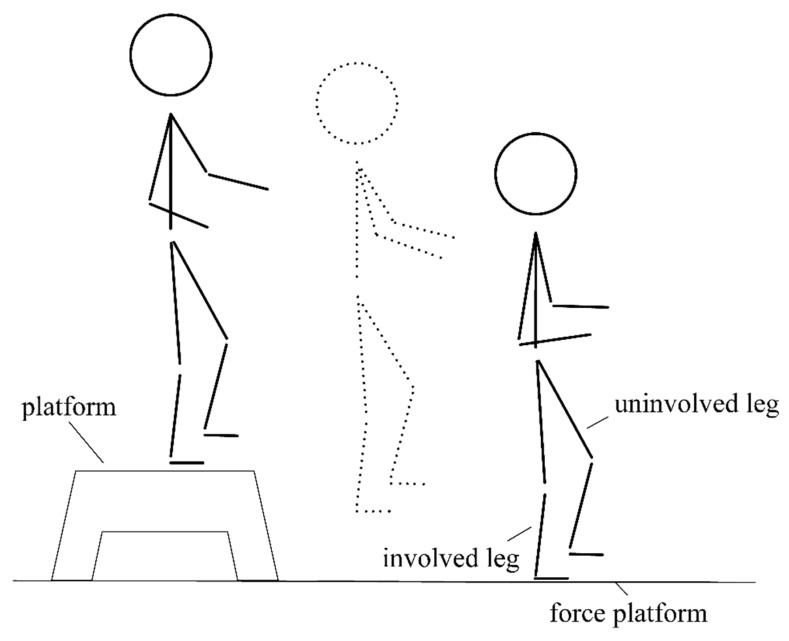
Schematic illustration of single-leg drop jump landing.

The single-leg drop jump landing test is usually used to examine balance impairments [34]. Participants were instructed to jump from a platform with a 15 cm height and land with the involved leg on a force platform, which is illustrated in Figure 2. The participant stood on the platform with the single involved leg. Upon hearing a verbal commencing order, the participant jumped on the force platform, landing with the involved leg. The participant were required to maintain balance as quickly as possible while their hands were on their hips. Failed trials were confirmed when the participant stood on the edge or out of the force platform. The trial was also excluded when the uninvolved leg touched the ground or hopped on the force platform. An entirely successful trial required the participant to keep balance with the involved leg for 20 s. Before the formal tests, all participants warmed up to adapt to the two-condition shoes and were instructed to conduct several pretests. During the interval of the test, sufficient rest of approximately 30 s [35] was provided for the participant in case fatigue influenced the result. The participant performed the experiment with different-condition shoes in random order. Three successful trials were required for each participant with these two footwear conditions.

### 2.4. Experiments Setup and Data Collection

A motion capture system with eight cameras (Vicon, Oxford Metrics Ltd., Oxford, UK) was used to record marker trajectories at a sampling frequency of 250 Hz, and one force platform was used to collect GRF at a sampling frequency of 1000 Hz (OR6, AMTI, Watertown, MA, USA) simultaneously. The Plug-in-Gait full-body human model in the Vicon was adopted to capture the trial data. Reflective markers were attached to the anatomical locations, and anthropometric parameters were measured for all participants. A total of 39 reflective markers for the Plug-in-Gait full-body human model were attached to the bone landmarks: front head over the temples, back head in a horizontal plane of the front head markers, 7th cervical vertebrae, 10th thoracic vertebrae, clavicle, sternum, right back, shoulder, arm, elbow, forearm, wrist, finger, anterior/posterior iliac spine, lateral femoral epicondyles, thigh, tibial, lateral ankle, toe, and heel [36]. 

A static calibration trial was conducted primarily, and single-leg drop jump landing trials were conducted subsequently. Three successful trials were recorded in the current study. The three-dimension marker trajectory data were filtered using a 4th-order Butterworth filter with a cut-off frequency of 7 Hz. The collected data of each participant were used to calculate COM. 

### 2.5. Data Reduction and Outcome Measures

**Figure 3:**
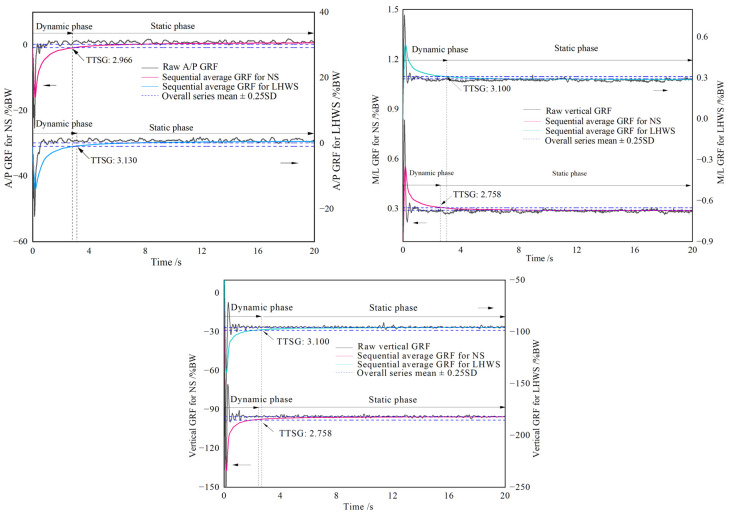
TTSG in three directions for single-leg jump trials for NS and LHWS. GRF: ground reaction force, TTSG: time to stabilization for ground reaction forces, TTSC: time to stabilization for the center of mass, NS: new shoes, LHWS: worn shoes with lateral attrition heel; SD: standard deviation, A/P: anteroposterior, M/L: mediolateral, BW: body weight.

Python 2.7 (Python Software Foundation, Beaverton, OR, USA) was used to perform data analysis. All GRF and COP data were filtered at 12 Hz with a bidirectional second-order Butterworth filter [37]. Then, a sequential averaging method was applied to calculate the time to stabilization for ground reaction forces (TTSG) as shown in Figure 3. The method calculated the cumulative average of all former data for each time point [17,20]. The threshold (as the dashed lines in Figure 3) was set to be the overall series mean ± 0.25 standard deviation (SD), which indicates a stable condition. TTSG was determined by the intersection of the sequential average line and the threshold, which is indicated in Figure 3. The static phase of the single-leg drop jump landing test was cropped from TTSG for all directions to the end of 20 s post impact, which excluded the dynamic phase. Outcome measures derived from COP were discriminative to assess static balance stability [38]. COP sway [39] was defined as the mean absolute distance between the instantaneous COP position and the average COP position during the static phase. COP SD [31] was defined as the overall SD of COP in a given direction during the static phase. 

**Figure 4:**
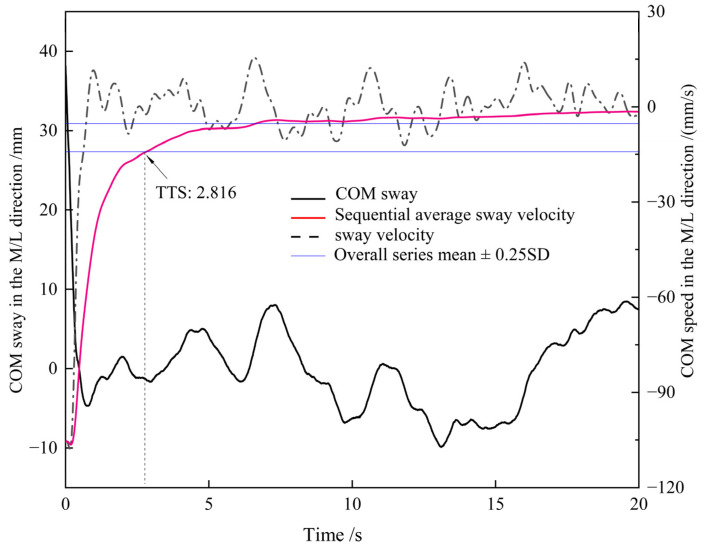
Interpretation diagram of COM velocity for a single trial. TTS: time to stabilization, COM: center of mass, SD: standard deviation, M/L: mediolateral.

The single-leg drop jump landing test was a transition procedure from the dynamic to the static phase. Outcome measures derived from force plates were unable to evaluate postural balance. As a result, trajectories of COM were adopted to evaluate the postural balance ability. After the initial heel contact following the drop jump, participants are required to restore stability as soon as possible, which altered the COM sway within a small range as represented in the black solid line in Figure 4. After the participants obtained postural stability, the instantaneous speed fluctuated within a small range as shown in Figure 4. In order to evaluate the ability of postural control, we calculated the TTS for COM speed. An interpretation diagram for the COM speed for a single trial is shown in Figure 4. We used the same threshold algorithm with TTS for GRF to determine the stabilization time for the COM. TTS has been proposed to be a discriminative method to detect stability for GRF [25,40,41]. In the current study, we applied this method to the COM speed due to a similar perturbation pattern. Both the GRF and COM speed presented a sudden decrease in the amplitude following the single-leg drop jump, and when stability was obtained, values fluctuated within a certain range The sequential averaging method was also applied to calculate the time to stabilization for the center of mass (TTSC). The threshold for postural stability was defined as the overall series mean ± 0.25 SD of COM sway velocity. Postural stability was confirmed when the sequential average sway velocity intersected with the threshold. 

### 2.6. Statistical Analysis

The statistical analysis was performed using SPSS (Version 22.0, IBM, Chicago, IL, USA). The mean outcome variables of three trials were calculated for each participant. Normal distribution was verified using the Shapiro–Wilk test with a significance level of 0.05. TTSG and TTSC in three directions and outcome variables derived from COP were tested for significance using paired t-tests with a significance level of 0.05 between the two conditions. Post hoc analyses were conducted to adjust *p*-values using the Bonferroni adjustment for TTSC and TTSG. Moreover, the effect size was represented with Cohen’s d, where 0.2 represents a small effect, 0.5 is a medium effect, and 0.8 reveals a large effect [42]. 

## 3. Results

Details of TTSG in three directions are reported in Table 1. TTSG in the A/P and vertical directions showed no significant differences between the NS group and the LHWS group. TTSG in the M/L direction in the LHWS group revealed a significant increase (*p =* 0.001, Cohen’s *d* = 0.970) compared to the NS group. With TTSG, the trial was divided into a dynamic phase and a static phase. Outcome measures of COP were calculated to determine the effects of the LHWS. For the static phase after TTSG, no group differences were found in maximum COP sway, mean COP sway, and COP SD, which were derived from COP for both the A/P and M/L directions in terms of the outcome measures derived from COP between the two conditions as shown in Table 2. TTSC derived from the COM of the two conditions is displayed in Table 3. The significant effect of TTSC showed similar results to TTSG between the two conditions. Only TTSC in the M/L direction revealed a slight significance (*p* = 0.016, Cohen’s *d* = 0.654) in the LHWS group when compared to the NS group. No significant differences were found in TTSC in the M/L and vertical directions between the two groups.

## 4. Discussion and Implications

The aim of this study was to examine the effects of LHWS on balance control ability and postural stability through single-leg drop jump landing tests. TTSG in three directions of GRF was calculated to quantify balance control ability using the sequential averaging method. In the M/L direction, a significant increase in TTSG was observed in the LHWS group when compared to the NS group. The outcome variables derived from COP showed no significant differences in the static phase in each direction. Additionally, TTSC was calculated to quantify postural control ability. Similarly, TTSC in the M/L direction was also significantly prolonged, and no significant changes were observed in the other two directions. 

The dynamic balance ability was assessed using measures of the force plate. Greater TTSG indicated impaired balance control ability [31]. TTSG in this study might be a little shorter than that in some previous studies [40], which could be caused by the lower height of the jump. In the M/L direction, TTSG increased significantly in the LHWS group. The increase indicated that participants need more time to recover their balance. LHWS was characterized by lateral heel degradation. The mediolateral asymmetry in the heel contributed to the larger changes in the GRF in the M/L direction. In contrast, TTSG in the A/P and vertical directions had limited changes. The worn region might account for the results as the worn part in the heel only occupied a small proportion in the A/P direction, which provided participants more space for posture regulation. On the other hand, the worn region in the M/L direction occupied a greater proportion, which resulted in less contact area for the foot [43]. A narrow support base would result in instability, which is interpreted as an increase in M/L TTSG. Furthermore, a previous study proposed that a longer TTSG has the potential to increase the risk of some injuries, such as anterior cruciate ligament injuries in collegiate athletes [44]. Worn shoes were also indicated to increase stance time and adapt kinematics [2]. Individuals with worn shoes might alter their normal patterns to regulate constant external loads. The longer TTSG in the LHWS group could be interpreted as more time being required to restore dynamic balance from perturbation. A significant increase in TTSG for the LHWS group was proposed to induce ankle instability [15]. Additionally, longer TTSG might reveal impairments in neuromuscular control and balance [20], which was proposed to be a risk of falling [25]. 

There were no significant differences in the static phase between the two groups based on the analysis of the outcome measures derived from the COP. The results indicated that worn outsoles did not affect balance control ability after the restoration of balance from the dynamic phase. With respect to the quantification of balance maintenance during the static phase, Fransz et al. [45] set the start of the static phase at 5 s post impact to evaluate balance. However, not all TTSG were less than 5 s [20], which might lead to misleading results. Consequently, the static phase was cropped from the TTSG to the end for each trial in the current study. Though body perturbation existed during the static phase, balance could be maintained through muscular strength. Additionally, the worn position focused on the lateral heel in this study. The COP might not lie in the worn region during the static phase, which could also account for the results. 

Outcome measures of GRF had deficiencies in the evaluation of postural balance that has high correlations with falls [46]. As a result, COM was used to assess the effects of LHWS on posture balance. Trajectories of COM possessed perturbation without absolute balance during single-legged standing. The variation range of COM trajectories in three directions remained fluctuated and thus could not determine the state when the dynamic phase transitioned to the static phase. Consequently, the COM velocity was calculated to determine the balance condition since the velocity would reach a small range of vibration around 0 after obtaining the postural control ability. The sequential averaging method was applied to COM velocities to calculate the time to stability for the COM (TTSC), which determined the postural control ability. Similarly, TTSC in the M/L direction showed a significant increase of 14.5% in the LHWS group when compared to the NS group. Lateral heel degradation impaired the support base, which led to a narrow support base. A narrow support base was highly related to weak frontal balance control, which made it difficult to maintain the COM within the support base [47]. The longer TTSC in the M/L direction could be interpreted as shoe degradation in the lateral heel narrowing the support base, which weakened the ability to achieve postural balance control and increased lateral fall possibilities. 

Both the TTSG and TTSC in the M/L direction increased significantly in the LHWS group, which implied that balance control ability and postural control ability were weakened in the M/L direction in the LHWS group. Consequently, it can be predicted that footwear with a lateral worn heel would increase balance risks in the M/L direction. Shoe degradation is inevitable over wearing time. In daily life, people usually neglect the degradation of footwear when they perform physical activities. However, LHWS could increase the instability of the wearer, based on the results of this study. The restoration time from oscillation during physical activities would increase for wearers with LHWS, which increases the possibility of fall risk. 

Some limitations in the study need to be mentioned. The sample size might not be large enough to consider the population variances. Although the participants practiced a few times under instructions before formal experiments, jump protocols might not be followed strictly for each trial. Considering the instability risk, only a 15 cm height platform was adopted to perform the jump tests. Another limitation might be the age of the participants. This study recruited young healthy adults. Therefore, the results might not be applied to older participants. The degradation of footwear might present different patterns, such as medial abrasion, lateral abrasion, and central abrasion. This study only adopted the most common pattern, which is the lateral heel abrasion, to discuss. Additionally, the interaction between worn shoes and individuals is a long-term mutual adaptation. In the current study, participants wore lab-provided shoes. We only investigated the immediate effects of worn shoes on balance. Therefore, the generalization of the current results should be noted. Future studies could focus on other worn patterns with the same experimental protocol. Nevertheless, the investigation revealed an implication that worn shoes predispose individuals to fall risks and other instability problems. 

## 5. Conclusions

Lateral-heel-attrition shoes had significant effects on balance control ability in the M/L direction. Both TTSG and TTSC increased in the LHWS group, which indicated that LHWS may lead to unstable injuries in the M/L direction. However, no significant differences between the LHWS group and NS group were observed in the other two directions. After the dynamic phase, LHWS had no effects on balance ability during the static phase according to the analyses of outcome measures of COP. Shoe degradation has the potential to increase injury risk. The results could also have implications for the evaluation of footwear.

## Figures and Tables

**Table 1:** Time to stabilization for ground reaction force(s).

Force Plate	NS Mean (SD)	LHWS Mean (SD)	*p*-Values	Cohen’s *d*
TTSG in A/P	2.939 (0.199)	2.974 (0.118)	0.409	0.206
TTSG in M/L	2.734 (0.232)	2.892 (0.248)	0.001 ^1^	0.970
Vertical TTSG	2.128 (0.257)	2.055 (0.298)	0.133	0.384

^1^ Significant difference at *p*-value < 0.05 when the LHWS group was compared with the NS group. Cohen’s d: 0.2 = small effect, 0.5 = medium effect, ≥0.8 = large effect. SD: standard deviation, A/P: anteroposterior, M/L: mediolateral, TTSG: time to stabilization for ground reaction forces, NS: new shoes, LHWS: worn shoes with lateral attrition heel.

**Table 2:** Outcome measures derived from the center of pressure.

Outcome Measures	NS Mean (SD)	LHWS Mean (SD)	*p*-Values	Cohen’s *d*
Maximum COP sway in A/P (mm)	71.015 (15.641)	74.173 (17.403)	0.404	0.208
Maximum COP sway in M/L (mm)	46.422 (17.738)	40.400 (18.373)	0.217	0.312
Mean COP sway in A/P (mm)	13.371 (3.424)	12.806 (2.938)	0.341	0.238
Mean COP sway in M/L (mm)	8.020 (1.853)	7.991 (1.374)	0.951	0.015
COP SD in A/P (mm)	18.220 (3.563)	17.487 (3.174)	0.476	0.177
COP SD in M/L (mm)	9.868 (2.074)	9.797 (1.557)	0.890	0.034

Cohen’s d: 0.2 = small effect, 0.5 = medium effect, ≥0.8 = large effect. COP: center of pressure, SD: standard deviation, A/P: anteroposterior, M/L: mediolateral, NS: new shoes, LHWS: worn shoes with lateral attrition heel.

**Table 3:** Time to stabilization for the center of mass (s).

Outcome Measures	NS Mean (SD)	LHWS Mean (SD)	*p*-Values	Cohen’s *d*
A/P TTSC	2.634 (0.389)	2.777 (0.314)	0.354	0.232
M/L TTSC	2.816 (0.705)	3.225 (0.550)	0.016 ^1^	0.654
Vertical TTSC	1.359 (0.529)	1.682 (1.244)	0.303	0.258

^1^ Significant difference at *p*-value < 0.05 when the LHWS group was compared with the NS. Cohen’s d: 0.2 = small effect, 0.5 = medium effect, ≥0.8 = large effect. SD: standard deviation, A/P: anteroposterior, M/L: mediolateral, TTSC: time to stabilization for the center of mass, NS: new shoes, LHWS: worn shoes with lateral attrition heel.

## Data Availability

Not applicable.

## References

[1] Hrysomallis C. (2007). Relationship between balance ability, training and sports injury risk. Sport. Med..

[2] Kong P.W., Candelaria N.G., Smith D.R. (2009). Running in new and worn shoes: A comparison of three types of cushioning footwear. Br. J. Sport. Med..

[3] Watanabe Y., Kawabe N., Mito K. (2021). The apex angle of the rocker sole affects the posture and gait stability of healthy individuals. Gait Posture.

[4] Hemler S.L., Pliner E.M., Redfern M.S., Haight J.M., Beschorner K.E. (2021). Effects of natural shoe wear on traction performance: A longitudinal study. Footwear Sci..

[5] Kwon Y.U. (2018). Static Postural Stability in Chronic Ankle Instability, an Ankle Sprain and Healthy Ankles. Int. J. Sport. Med..

[6] Asplund C.A., Brown D.L. (2005). The running shoe prescription: Fit for performance. Phys. Sport..

[7] Sole C.C., Milosavljevic S., Sole G., Sullivan S.J. (2010). Exploring a model of asymmetric shoe wear on lower limb performance. Phys. Ther. Sport.

[8] Sundaram V.H., Hemler S.L., Chanda A., Haight J.M., Redfern M.S., Beschorner K.E. (2020). Worn region size of shoe outsole impacts human slips: Testing a mechanistic model. J. Biomech..

[9] Chinn L., Hertel J. (2010). Rehabilitation of ankle and foot injuries in athletes. Clin. Sport. Med..

[10] Ringhof S., Stein T. (2018). Biomechanical assessment of dynamic balance: Specificity of different balance tests. Hum. Mov. Sci..

[11] Gribble P.A., Robinson R.H. (2009). Alterations in Knee Kinematics and Dynamic Stability Associated with Chronic Ankle Instability. J. Athl. Train..

[12] Huurnink A., Fransz D.P., Kingma I., de Boode V.A., Dieen J.H.V. (2019). The assessment of single-leg drop jump landing performance by means of ground reaction forces: A methodological study. Gait Posture.

[13] Fransz D.P., Huurnink A., Kingma I., Verhagen E.A., van Dieen J.H. (2013). A systematic review and meta-analysis of dynamic tests and related force plate parameters used to evaluate neuromusculoskeletal function in foot and ankle pathology. Clin. Biomech..

[14] Wikstrom E.A., Tillman M.D., Smith A.N., Borsa P.A. (2005). A new force-plate technology measure of dynamic postural stability: The dynamic postural stability index. J. Athl. Train..

[15] Zech A., Argubi-Wollesen A., Rahlf A.L. (2015). Minimalist, standard and no footwear on static and dynamic postural stability following jump landing. Eur. J. Sport Sci..

[16] Sung P.S. (2016). The ground reaction force thresholds for detecting postural stability in participants with and without flat foot. J. Biomech..

[17] Wikstrom E.A., Tillman M.D., Borsa P.A. (2005). Detection of dynamic stability deficits in subjects with functional ankle instability. Med. Sci. Sport. Exerc..

[18] Bowser B.J., Rose W.C., McGrath R., Salerno J., Wallace J., Davis I.S. (2017). Effect of Footwear on Dynamic Stability during Single-leg Jump Landings. Int. J. Sport. Med..

[19] Letafatkar A., Mantashloo Z., Moradi M. (2018). Comparison the time to stabilization and activity of the lower extremity muscles during jump-landing in subjects with and without Genu Varum. Gait Posture.

[20] Fransz D.P., Huurnink A., de Boode V.A., Kingma I., van Dieen J.H. (2015). Time to stabilization in single leg drop jump landings: An examination of calculation methods and assessment of differences in sample rate, filter settings and trial length on outcome values. Gait Posture.

[21] Clark R.A., Bryant A.L., Pua Y., McCrory P., Bennell K., Hunt M. (2010). Validity and reliability of the Nintendo Wii Balance Board for assessment of standing balance. Gait Posture.

[22] Robbins S.M., Caplan R.M., Aponte D.I., St-Onge N. (2017). Test-retest reliability of a balance testing protocol with external perturbations in young healthy adults. Gait Posture.

[23] Hrysomallis C. (2011). Balance ability and athletic performance. Sport. Med..

[24] Federolf P.A., Roos L., Nigg B. (2012). The effect of footwear on postural control in bipedal quiet stance. Footwear Sci..

[25] Charles S.C., Stephan M., Gisela S., Sullivan S.J. (2018). Dynamic postural stability is more variable barefoot than in footwear in healthy individuals. Footwear Sci..

[26] Huang M., Yick K.L., Ng S.P., Yip J., Cheung R.T. (2020). The effect of support surface and footwear condition on postural sway and lower limb muscle action of the older women. PLoS ONE.

[27] Talarico M.K., Lynall R.C., Mauntel T.C., Weinhold P.S., Padua D.A., Mihalik J.P. (2017). Static and dynamic single leg postural control performance during dual-task paradigms. J. Sport. Sci..

[28] Benvenuti F., Mecacci R., Gineprari I., Bandinelli S., Benvenuti E., Ferrucci L., Baroni A., Rabuffetti M., Hallett M., Dambrosia J.M. (1999). Kinematic characteristics of standing disequilibrium: Reliability and validity of a posturographic protocol. Arch. Phys. Med. Rehabil..

[29] Corriveau H., Hebert R., Raiche M., Dubois M.F., Prince F. (2004). Postural stability in the elderly: Empirical confirmation of a theoretical model. Arch. Gerontol. Geriatr..

[30] Taunton J.E., Ryan M.B., Clement D.B., McKenzie D.C., Lloyd-Smith D.R., Zumbo B.D. (2003). A prospective study of running injuries: The Vancouver Sun Run “In Training” clinics. Br. J. Sport. Med..

[31] Ross S.E., Guskiewicz K.M., Gross M.T., Yu B. (2009). Balance measures for discriminating between functionally unstable and stable ankles. Med. Sci. Sport. Exerc..

[32] Finestone A.S., Petrov K., Agar G., Honig A., Tamir E., Milgrom C. (2012). Pattern of outsole shoe heel wear in infantry recruits. J. Foot Ankle Res..

[33] Saito S., Muraki S., Tochihara Y. (2007). Effects of worn-out soles on lower limb stability, shock absorption and energy cost during prolonged walking. J. Physiol. Anthropol..

[34] Caulfield B.M., Garrett M. (2002). Functional Instability of the Ankle: Differences in Patterns of Ankle and Knee Movement Prior To and Post Landing in a Single Leg Jump. Int. J. Sport. Med..

[35] Wright C.J., Arnold B.L., Ross S.E. (2016). Altered Kinematics and Time to Stabilization During Drop-Jump Landings in Individuals With or Without Functional Ankle Instability. J. Athl. Train..

[36] Panero E., Digo E., Ferrarese V., Dimanico U., Gastaldi L. Multi-Segments Kinematic Model of the Human Spine during Gait. Proceedings of the 2021 IEEE International Symposium on Medical Measurements and Applications (MeMeA).

[37] Huurnink A., Fransz D.P., Kingma I., van Dieen J.H. (2013). Comparison of a laboratory grade force platform with a Nintendo Wii Balance Board on measurement of postural control in single-leg stance balance tasks. J. Biomech..

[38] Doyle R.J., Hsiao-Wecksler E.T., Ragan B.G., Rosengren K.S. (2007). Generalizability of center of pressure measures of quiet standing. Gait Posture.

[39] Thompson C., Schabrun S., Romero R., Bialocerkowski A., van Dieen J., Marshall P. (2017). Factors Contributing to Chronic Ankle Instability: A Systematic Review and Meta-Analysis of Systematic Reviews. Sport. Med..

[40] Malmir K., Olyaei G.R., Talebian S., Jamshidi A.A., Ganguie M.A. (2019). Effects of Peroneal Muscles Fatigue on Dynamic Stability Following Lateral Hop Landing: Time to Stabilization Versus Dynamic Postural Stability Index. J. Sport Rehabil..

[41] Garcia-Masso X., Skypala J., Jandacka D., Estevan I. (2019). Reliability of a new analysis to compute time to stabilization following a single leg drop jump landing in children. PLoS ONE.

[42] Molina-Molina A., Latorre-Roman P.A., Mercado-Palomino E., Delgado-Garcia G., Richards J., Soto-Hermoso V.M. (2022). The effect of two retraining programs, barefoot running vs increasing cadence, on kinematic parameters: A randomized controlled trial. Scand. J. Med. Sci. Sport..

[43] Ross S.E., Guskiewicz K.M., Yu B. (2005). Single-Leg Jump-Landing Stabilization Times in Subjects With Functionally Unstable Ankles. J. Athl. Train..

[44] DuPrey K.M., Liu K., Cronholm P.F., Reisman A.S., Collina S.J., Webner D., Kaminski T.W. (2016). Baseline Time to Stabilization Identifies Anterior Cruciate Ligament Rupture Risk in Collegiate Athletes. Am. J. Sport. Med..

[45] Fransz D.P., Huurnink A., Kingma I., van Dieen J.H. (2014). How does postural stability following a single leg drop jump landing task relate to postural stability during a single leg stance balance task?. J. Biomech..

[46] Yang F., Anderson F.C., Pai Y.C. (2008). Predicted threshold against backward balance loss following a slip in gait. J. Biomech..

[47] Chien H.L., Liu M.W., Lu T.W., Kuo C.C., Chung P.C. (2014). Inter-joint sharing of total support moments in the lower extremities during gait in narrow-heeled shoes of different heights. Ergonomics.

